# Non-Invasive Prediction of Atrial Fibrosis Using a Regression Tree Model of Mean Left Atrial Voltage

**DOI:** 10.3390/biomedicines13081917

**Published:** 2025-08-06

**Authors:** Javier Ibero, Ignacio García-Bolao, Gabriel Ballesteros, Pablo Ramos, Ramón Albarrán-Rincón, Leire Moriones, Jean Bragard, Inés Díaz-Dorronsoro

**Affiliations:** 1 Cardiology and Cardiac Surgery Department, Clínica Universidad de Navarra, Avenida Pio XII 36, 31008 Pamplona, Spain; igarciab@unav.es (I.G.-B.); pablo80ra@hotmail.com (P.R.); ralbarran@unav.es (R.A.-R.); adiazdo@unav.es (I.D.-D.); 2 Cardiology and Cardiac Surgery Department, Hospital Universitario de Navarra, C. de Irunlarrea 3, 31008 Pamplona, Spain; 3 Cardiology Department, Hospital Reginal Universitario de Málaga, Avenida de Carlos Haya 84, 29010 Málaga, Spain; gabrielballesteros83@hotmail.com; 4 Physics and Applied Mathematics Department, Universidad de Navarra, Calle de Irunlarrea 1, 31008 Pamplona, Spain; lmoriones.1@alumni.unav.es (L.M.); jbragard@unav.es (J.B.); 5 Data Science and Artificial Intelligence Institute (DATAI), Universidad de Navarra, Calle Universidad 6, 31009 Pamplona, Spain

**Keywords:** atrial fibrosis, atrial cardiomyopathy, echocardiography, machine learning

## Abstract

**Background**: Atrial fibrosis is a key contributor to atrial cardiomyopathy and can be assessed invasively using mean left atrial voltage (MLAV) from electroanatomical mapping. However, the invasive nature of this procedure limits its clinical applicability. Machine learning (ML), particularly regression tree-based models, may offer a non-invasive approach for predicting MLAV using clinical and echocardiographic data, improving non-invasive atrial fibrosis characterisation beyond current dichotomous classifications. **Methods**: We prospectively included and followed 113 patients with paroxysmal or persistent atrial fibrillation (AF) undergoing pulmonary vein isolation (PVI) with ultra-high-density voltage mapping (uHDvM), from whom MLAV was estimated. Standardised two-dimensional transthoracic echocardiography was performed before ablation, and clinical and echocardiographic variables were analysed. A regression tree model was constructed using the Classification and Regression Trees—CART-algorithm to identify key predictors of MLAV. **Results**: The regression tree model exhibited moderate predictive accuracy (R^2^ = 0.63; 95% CI: 0.55–0.71; root mean squared error = 0.90; 95% CI: 0.82–0.98), with indexed minimum LA volume and passive emptying fraction emerging as the most influential variables. No significant differences in AF recurrence-free survival were found among MLAV tertiles or model-based generated groups (log-rank *p* = 0.319 and *p* = 0.126, respectively). **Conclusions**: We present a novel ML-based regression tree model for non-invasive prediction of MLAV, identifying minimum LA volume and passive emptying fraction as the most significant predictors. This model offers an accessible, non-invasive tool for refining atrial cardiomyopathy characterisation by reflecting the fibrotic substrate as a continuum, a crucial advancement over existing dichotomous approaches to guide tailored therapeutic strategies.

## 1. Introduction

Artificial intelligence, particularly machine learning (ML), has become an increasingly valuable tool in biomedical research and clinical decision-making [1]. By leveraging large and heterogeneous datasets, ML algorithms can identify complex, nonlinear patterns that may be overlooked by traditional statistical methods, thereby improving the prediction of clinical variables and outcomes. Among the various ML techniques, regression tree-based models stand out for their balance of predictive performance and interpretability, making them particularly suitable for clinical use due to their robustness and ease of implementation [1,2]. These models generate intuitive decision rules that facilitate user-friendly integration into routine clinical workflows.

Atrial fibrosis is a key component of atrial cardiomyopathy and plays a pivotal role in the pathophysiology of atrial fibrillation (AF) [3]. Beyond its association with AF recurrence, atrial fibrosis contributes to adverse atrial remodelling, increased thromboembolic risk, and the progression from paroxysmal to persistent AF [4]. Electroanatomical mapping is considered the gold standard for identifying atrial fibrosis because it can identify low voltage areas (<0.50 mV), conventionally interpreted as dense fibrotic tissue. However, the use of this specific cutoff remains controversial, as voltage amplitude can be influenced by various technical and anatomical factors [5].

Mean left atrial voltage (MLAV) has emerged as a robust electroanatomical predictor of AF recurrence after pulmonary vein isolation (PVI), as it captures both dense scars and interstitial fibrosis [6]. Its relevance as a surrogate marker of atrial fibrosis is supported by correlations with structural and biochemical fibrosis markers. Additionally, exploratory studies suggest that MLAV, in combination with slope voltage, may outperform low-voltage area extension alone in predicting AF recurrences [6].

Despite its value, the invasive nature of electroanatomical mapping limits its applicability in routine clinical practice. Therefore, developing a non-invasive method to estimate MLAV could facilitate early detection of advanced atrial cardiomyopathy and support personalised therapeutic decisions. To this end, imaging modalities such as echocardiography and MRI have been explored, although their correlation with electroanatomical findings has been modest [7,8]. In parallel, several ML models have been developed to predict the presence of low-voltage areas, but most adopt a dichotomous approach—such as presence versus absence of fibrosis [9]—which oversimplifies the continuous nature of atrial fibrotic burden and may limit clinical applicability.

To address these limitations, we developed a regression tree model to predict MLAV as a continuous variable, enabling a more refined and quantitative assessment of atrial fibrosis. This approach represents a significant advancement over traditional dichotomous classifications, with the potential to guide more personalised management strategies based on the degree of atrial remodelling. A model based on routinely available clinical and echocardiographic variables may facilitate wider clinical implementation.

We present an exploratory ML-based regression tree model for predicting MLAV, offering clinicians an accessible tool for enhancing atrial cardiomyopathy characterisation.

## 2. Materials and Methods

**Figure 1:**
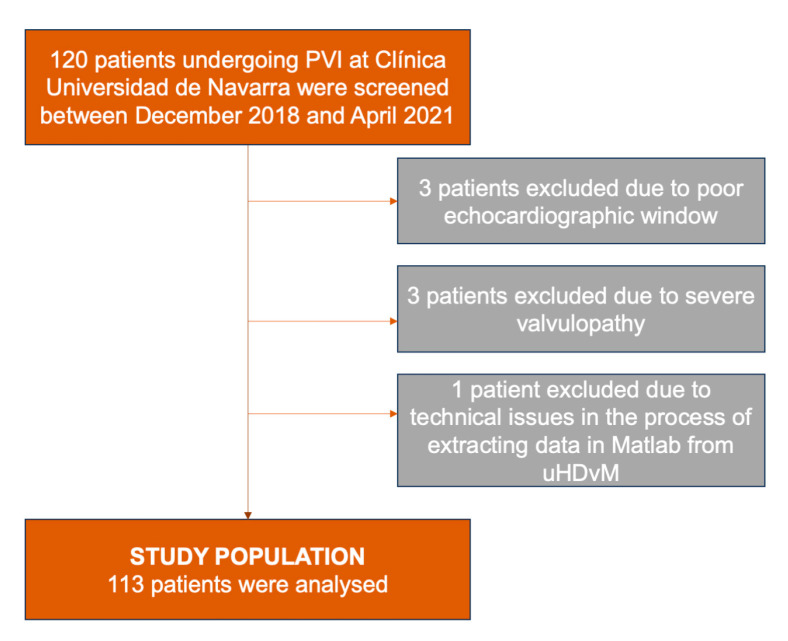
Flowchart showing the selection process of the study population. PVI, pulmonary vein isolation; uHDvM, ultra-high-density voltage mapping.

We prospectively screened 120 patients with paroxysmal or persistent AF who were scheduled for PVI using an ultra-high-density voltage mapping (uHDvM) system at Clínica Universidad de Navarra (CUN; Pamplona, Spain) between December 2018 and April 2021. Clinical follow-up was concluded in December 2023. Figure 1 illustrates a flowchart showing the selection process of the study population. The study protocol was approved by the Institutional Ethics Committee, and the study was conducted according to the principles of the Declaration of Helsinki. Written informed consent was obtained from all participants. Data supporting the findings of this study are available from the corresponding author upon reasonable request.

### 2.1. Clinical Variables

Clinical characteristics—including age, sex, body mass index, traditional cardiovascular risk factors, and relevant comorbidities—were retrospectively extracted from the electronic medical records of patients treated at CUN.

Baseline functional status was evaluated using the European Heart Rhythm Association (EHRA) classification, while thromboembolic risk was assessed using the CHA2DS2-VASc score. AF type (paroxysmal or persistent) was categorised according to the latest European guidelines [10]. In patients with a history of PVI, the number of procedures and techniques employed was also recorded.

### 2.2. Echocardiographic Protocol

Transthoracic echocardiography was performed immediately prior to PVI using a GE Vivid™ E95 system (General Electric, Chicago, IL, USA). Image acquisition followed current guidelines [11,12], with a standardised minimum image dataset acquired at a minimum frame rate of 55 Hz over five cardiac cycles. Multiplanar echocardiographic image acquisition was performed using a dual-plane (biplane) imaging modality, enabling simultaneous orthogonal views of the left atrium. For those patients in AF at the time of imaging acquisition, left atrial volumes were calculated as the average of five consecutive cardiac cycles. Offline measurements were performed using IntelliSpace Cardiovascular (v6.10, Philips Medical Systems, Best, The Netherlands) and Tomtec Arena (v.TTA2.50, TomTec Imaging Systems, Unterschleissheim, Germany). To assess intra- and interobserver variability, 20 patients were randomly selected and independently reanalysed in a blinded fashion by the same echocardiography expert and a second reviewer.

Left ventricular (LV) and LA dimensions were measured using 2D techniques. LV volumes and ejection fraction were calculated using the Simpson biplane method. LV global longitudinal strain (GLS) and LA strain were analysed offline using vendor-independent software (Tomtec Arena v. TTA2.50). The QRS complex onset was used as the reference point for the cardiac cycle. LV systolic and diastolic function, right ventricular function, and valvular disease were assessed and classified according to current guidelines [11]. All volumetric values were indexed to the body surface area.

LA volumes were measured using the disc summation method and included maximum (LAmax), minimum (LAmin), and pre-atrial contraction volume (LApreA). The following functional indices were derived: LA total emptying fraction (LAEF, %) (Lamax − LAmin)/Lamax; LA passive emptying fraction (LApEF, %) (Lamax − LApreA)/Lamax; and LA active ejection fraction (LAactEF, %) (LApreA − LAmin)/LApreA. The LA sphericity index was calculated as previously described [13,14].

### 2.3. Ultra-High-Density Mapping and Quantitative Map Analysis

The mapping and ablation procedures were performed as previously described [15,16]. Post-processing of the maps also followed established methodologies [6]. If the patient was in AF, electrical cardioversion was performed before starting the procedure. For each patient, one ultra-high-density voltage map of the LA in sinus rhythm was obtained before ablation. A conventional decapolar diagnostic catheter was placed in the coronary sinus for pacing, as an electric and anatomical reference for impedance tracking. Access to the left atrium was performed by means of double transseptal puncture for the basket-type mapping catheter (IntellaMap Orion, Boston Scientific Corporation, Burlington, MA, USA) and for the ablation catheter (Intella Nav OI or Blazer OI, Boston Scientific Corporation, Marlborough, MA, USA). Both catheters were positioned using dedicated steerable sheaths (Agilis; St Jude Medical, St Paul, MN, USA). Mapping of the left atrium was conducted with an IntellaMap Orion catheter during atrial pacing from the proximal coronary sinus, with a cycle length of 550 ms. The appropriate beats and electrograms were automatically selected by the system in accordance with predefined criteria. The initial criteria were stability of cycle length, with a tolerance of ±10 ms; a propagation reference, with a tolerance of ±5 ms; a respiratory cycle accepting only beats during the exhalation phase; Motion = 1 mm; Stability = 0.25; and Tracking = 3. Only points located within 2 mm of the external surface of the map were considered for analysis. In all cases, special attention was given to acquiring a very high-density point map at the level of the venous antra and the left ridge by slowly moving and rotating the Orion catheter. Exploration of the septal region was performed by gently withdrawing the fully expanded Orion catheter until resistance from the interatrial septum was encountered. Then, PVs and LA appendage were digitally excluded from the electroanatomical map. In the redo procedures, previous encircling ablation lines—as identified by the surgeon using the activation maps— were also removed to avoid underestimation of MLAV, as the objective was to assess native fibrosis rather than scar tissue generated resulting from prior procedures.

**Figure 2:**
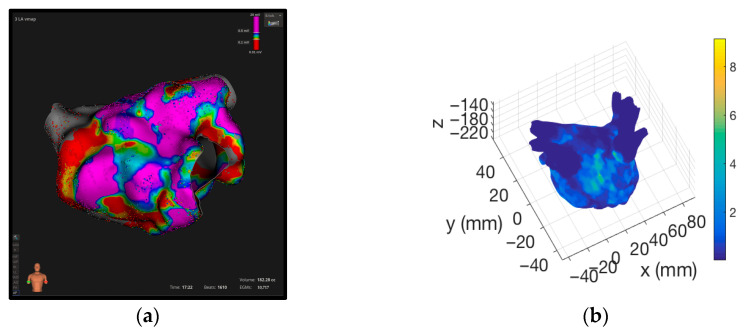
Electrophysiological study of the left atrium. (**a**) Ultra-high-density voltage mapping; (**b**) MatLab reconstruction.

The resulting voltage maps were then converted to a format compatible with MATLAB (v2018A, The MathWorks Inc., Natick, MA, USA) for further quantitative analysis (Figure 2). Each map consisted of a 3D geometrical surface defined by a triangular mesh. The mean number of triangle faces was 11,037.00 ± 2211.00, with an average area of approximately 1 mm^2^, following a near-Gaussian distribution. Bipolar voltage values (in mV) were assigned to each vertex. For each map, the spatial average of all bipolar voltages across the LA surface was calculated.

After mapping, PVI was performed in all cases. De novo PVI procedures were performed by standard point-by-point ablation, creating wide ablation circles around the PVs, while redo procedures were performed by the analysis of the activation maps and focal ablation at the reconnection gaps. In all cases, isolation was confirmed with the insertion of an Orion catheter within the PVs and its subsequent expansion. In the event of confirmation of entrance block, the pacing was performed from the equatorial electrodes of the Orion catheter to confirm exit block. In all cases and for each of the isolated veins, an intravenous bolus of adenosine was administered with the Orion catheter inside the vein, and a focal ablation was performed in the event of an observed reconnection. Isolation was confirmed in all cases at a minimum of 20 min after the last radiofrequency application.

### 2.4. Statistical Analysis

We did not perform a formal a priori sample size calculation, consistent with the exploratory nature of this study, which aimed to identify key predictors and demonstrate proof of concept for a non-invasive model to predict mean left atrial voltage (MLAV). The cohort of 113 patients, along with the observed event rate, was considered sufficient for the development and internal validation of an interpretable regression tree.

Regression tree models obtained using the Classification and Regression Trees algorithm (CART), implemented through the RPART package in R, were employed to identify clinical and echocardiographic variables that may influence the prediction of subjects’ mean voltage. CART was intentionally selected for its high interpretability and clinical transparency, allowing for the generation of intuitive decision rules that support clinical integration. Due to moderate sample size, we prioritised model interpretability over maximum predictive performance. Although more complex ML methods (e.g., Random Forest or XGBoost) may offer higher accuracy, they often do so at the cost of interpretability. As our intent was to establish a clinically intuitive proof-of-concept model, we did not pursue ensemble approaches at this stage.

CART is a non-parametric method that does not assume normality or linearity of the predictor variables or residuals, which distinguishes it from traditional statistical approaches such as linear regression. For variables with less than 10% missing data, a complete case analysis was performed. Accordingly, variables with more than 10% missing values were excluded from the modelling process, resulting in the exclusion of 12.01% (7/58) of all candidate variables. These data were not missing due to collection errors but rather because their availability was inherently limited by the fact that the patient was in atrial fibrillation at the time of the echocardiographic examination (e.g., left atrium preA volume) or spectral Doppler envelopes were incomplete. No imputation techniques were applied to preserve the integrity of the original data. Tables S3 and S4 list all variables analysed. Predictive values were obtained using a training set (80% of the cases) and evaluated on a test set (20% of the cases) using cross-validation. To minimise overfitting, we applied cost-complexity pruning during model construction, with complexity parameters optimised using 10-fold cross-validation. We did not perform bootstrap resampling, as internal validation was deemed sufficient for this exploratory phase. By including rhythm status at the moment of echocardiography, the model partially accounts for its potential confounding effect through hierarchical partitioning, even though no explicit adjustment coefficients are derived, as would be the case in parametric models. Rhythm-specific subgroup models were explored. All modelling and statistical procedures were conducted in accordance with the TRIPOD (Transparent Reporting of a multivariable prediction model for Individual Prognosis Or Diagnosis) guidelines to ensure transparency and reproducibility of the study.

Additionally, standard statistical analyses were conducted to characterise the study population and explore group-level differences. Descriptive statistics were reported as counts and percentages for categorical variables and as means ± standard deviations for continuous variables. Normality was assessed via Q–Q plots, skewness and kurtosis, and the Shapiro–Wilk test. Tables S1 and S2 summarise the feasibility of the echocardiographic parameters and their intraclass correlation coefficients.

Comparisons of categorical variables were performed using the chi-square test or Fisher’s exact test, as appropriate. Before applying analysis of variance (ANOVA), the assumption of homogeneity of variances was evaluated using Bartlett’s test. In cases where this assumption was not met or the residuals did not follow a normal distribution, the Kruskal–Wallis test was applied as a non-parametric alternative.

Standardised differences were calculated to assess the magnitude of differences between groups for both continuous and categorical variables, independently of sample size. Cohen’s *d* was used for continuous variables, and Cramér’s *V* was used for categorical variables, as appropriate. Effect sizes were interpreted using conventional thresholds: for Cohen’s *d*, (I) negligible if |<0.20|, (S) small if |0.20–0.49|, (M) moderate if |0.50–0.79|, and (L) large if|≥0.80|; for Cramér’s *V*, (I) negligible if |<0.10|, (S) small if |0.10–0.29|, (M) moderate if |0.30–0.49|, and (L) large if |≥0.50|.

Post hoc analyses were performed to determine specific group differences, with Bonferroni-adjusted *p*-values (*p*’). Table S5 presents a detailed report of the post hoc analysis. Kaplan–Meier survival curves and log rank test were used to evaluate time-to-event outcomes.

All statistical analyses were performed using STATA (version 18.0; StataCorp LLC, College Station, TX, USA) and RStudio version (version 4.3.2; R Foundation for Statistical Computing, Vienna, Austria). The regression tree model was implemented using the rpart package (version 4.1.19), and data preprocessing was conducted using the tidyverse ecosystem.

## 3. Results

### 3.1. Study Population: Clinical and Electroanatomical Characteristics

The final study cohort included 113 patients with paroxysmal or persistent AF who were admitted for PVI. The mean age at the time of PVI was 63.69 ± 10.19 years, and 72.57% of patients were male. The mean interval between AF diagnosis and PVI was 40.99 ± 52.78 months. Paroxysmal AF was present in 70.80% of the patients, while 61.06% of the patients were in sinus rhythm upon entry to the electrophysiology laboratory. Thirteen patients (11.50%) had previously undergone PVI. The mean EHRA class was 1.95 ± 0.37, and the mean CHA_2_DS_2_-VASc score was 1.82 ± 1.43. Patients were followed up until December 2023, with a mean follow-up duration of 33.84 ± 16.40 months and a median of 37 months. AF recurrence was identified using either standard ECG or 24 h Holter monitoring. Fifty-two patients (46.85%) experienced recurrence during follow-up.

Pre-ablation uHDvM was successfully performed in all patients. The mean number of mapping points per patient was 11,037.74 ± 2211.89, whereas the overall mean voltage was 1.41 ± 0.85 mV. Table 1 presents a detailed summary of clinical and electroanatomical variables.

### 3.2. Echocardiographic Characteristics

All patients had normal LV dimensions, with a mean LVEF of 58.38% ± 7.32%. The mean LA anteroposterior diameter was 42.32 ± 6.99 mm. LA volumes increased across all atrial phases. The mean LAmax, LAmin, and LApreA volumes were 41.60 ± 11.97, 26.39 ± 12.48, and 27.09 ± 9.79 mL/m^2^, respectively. The mean LAEF, LApEF, and LAactEF were 38.59% ± 16.55%, 57.73% ± 35.10%, and 30.11% ± 17.10%, respectively. Reservoir strain (LASres), conduit strain (LAScd), and contraction strain (LASct) were 25.36 ± 13.13%, 17.25 ± 8.10%, and 13.05 ± 5.64%, respectively. The mean E-wave peak velocity (VmaxE) and the mean E/E′ ratio were 81.56 ± 21.19 cm/s and 8.26 ± 3.34, respectively. Table 2 presents an extended report of the echocardiographic variables.

### 3.3. Mean Voltage Regression Tree

**Figure 3:**
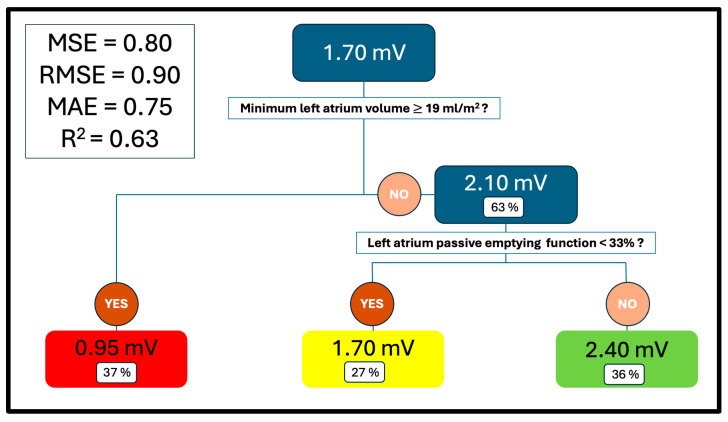
Mean voltage regression tree. LA, left atria; Vol, volume; model performance metrics: MSE, Mean Squared Error; RMSE, Root Mean Squared Error, MAE, Mean Absolute Error; R^2^, Coefficient of Determination.

The generated regression model (Figure 3) divided the population into three groups according to the predictor variables, assigning each group a mean predicted value of MLAV: 0.95 mV, 1.70 mV, and 2.40 mV. These values represent the model’s average predictions for each group of patients with similar characteristics. The model exhibited acceptable predictive performance, with a mean squared error of 0.80, root mean squared error of 0.90 (95% CI: 0.82–0.98), and mean absolute error of 0.75. The coefficient of determination (R^2^) was 0.63 (CI: 0.55–0.71), indicating that the model accounted for 63.00% of the variance in the outcome variable.

To assess the potential impact of rhythm status during echocardiography, additional subgroup analyses were performed for patients in atrial fibrillation and those in sinus rhythm. However, the rhythm-specific models demonstrated lower predictive performance (R^2^ = −1.10 for AF and R^2^ = −1.81 for sinus rhythm), suggesting that the global model provided more stable and accurate estimations, likely due to greater statistical power and reduced variance in the overall cohort. Table S6 and Figures S1 and S2 provide a detailed summary of these subanalyses.

Decision tree analysis identified the indexed minimum LA volume as the primary splitting variable. Among patients with values <19.00 mL/m^2^, further stratification by passive emptying fraction revealed that those with values <33.00% had a higher mean outcome (2.40 mV) than those with values ≥33.00% (1.70 mV). These results suggest that both the indexed minimum LA volume and the passive emptying fraction are relevant predictors in the model.

### 3.4. Comparative Analysis Between Groups

Clinical and echocardiographic variables were compared across the groups generated by the regression tree. Table 1 and Table 2 present the results. Patients in the “2.40 mV” group were younger (*p*’ = 0.006), had a higher prevalence of paroxysmal AF (*p*’ = 0.009) and lower CHA_2_DS_2_-VASc scores (*p*’ = 0.022), and exhibited a proportionally lower incidence of AF recurrence after PVI (*p*’ = 0.039) compared with those in the ‘0.95 mV’ mean voltage group. Moreover, patients in the “1.70 mV” and “2.40 mV” groups were more frequently in AF at the time of echocardiographic assessment (both *p*’ < 0.001), had thicker LV septal walls (*p*’ = 0.013 and 0.001, respectively), and exhibited larger LA and poorer LA function—including LA strain—regardless of the timing within the atrial cycle, than those in the “0.95” group. No additional significant clinical or echocardiographic differences were observed. Table S6 provides a detailed report of the post hoc analysis.

### 3.5. Survival Analysis

**Figure 4:**
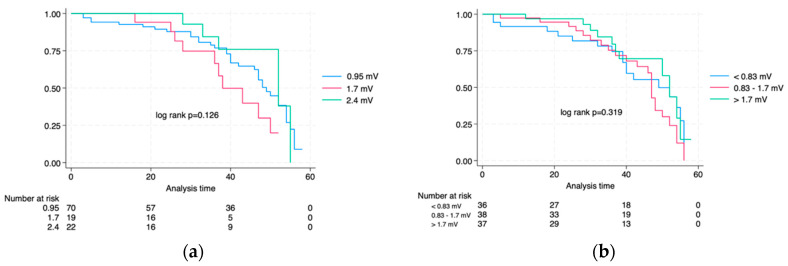
Kaplan-Meier curves. Time-to-event analysis. (**a**) Regression tree model. (**b**) MLAV tertiles.

Of the 113 patients analysed, 111 completed the follow-up at our centre The mean, median, and maximum follow-ups were 34 months, 37 months, and 58 months, respectively. Among these 111 patients, 52 (46.84%) experienced AF recurrence by the end of the study. The unadjusted AF-free survival rates at 1 and 3 years were 95.00% and 81.00%, respectively, with a median AF recurrence-free survival of 49 months. No significant differences were found in the time-to-event analysis between tertiles of MLAV (Figure 4, log rank *p*’ = 0.319) or between the groups generated by the regression tree model (Figure 4, log rank *p*’ = 0.126).

## 4. Discussion

To the best of our knowledge, this is the first published ML-based model to predict MLAV and, consequently, atrial fibrosis, a hallmark of atrial cardiomyopathy. MLAV is an electrophysiological parameter that captures both dense and interstitial fibrosis, offering a more comprehensive assessment of atrial structural remodelling. Its correlation with established biomarkers of atrial fibrosis has been previously demonstrated [17], and it has shown, in combination with slope voltage, a greater predictive value for AF recurrence after PVI than conventional parameters, such as maximum LA volume or the extent of dense atrial scarring identified via electroanatomical mapping [6].

Previous studies have explored the correlation between various imaging parameters—both echocardiographic and cardiac MRI-derived—and atrial fibrosis as quantified by electroanatomical voltage mapping. These studies generally fall into two categories: those proposing specific cutoff values to dichotomise patients into “fibrotic” and “non-fibrotic” groups based on AF recurrence risk, and those assessing individual imaging parameters in correlation with fibrosis burden, often with limited predictive accuracy [7,8]. However, atrial fibrosis and atrial cardiomyopathy are best understood as a continuous process rather than a binary condition. Therefore, for both the characterisation of atrial cardiomyopathy and the initial risk stratification of patients with AF, estimating a continuous, approximate value of the mean atrial voltage may provide a more clinically relevant assessment.

The incorporation of ML into cardiovascular imaging has marked a significant paradigm shift, allowing for the identification of complex, nonlinear patterns that traditional methods often underestimate [1]. Regression trees stand out for their robust predictive capabilities and clinician-friendly interpretability [2]. In our model, the most reliable predictors of atrial fibrosis were minimum LA volume and passive atrial emptying fraction.

While the regression tree model achieved an R^2^ of 0.63 and an RMSE of 0.90 mV, these metrics must be interpreted within the clinical context of MLAV and its variability. In our study cohort, the mean MLAV was 1.41 mV, with a standard deviation of 0.85 mV, suggesting that most values ranged between approximately 0.2 mV and 3.1 mV. Therefore, a prediction error of 0.90 mV represents roughly one quarter of the observed variation. Although this may limit the model’s utility for precise individual-level predictions, it remains informative for broader, non-invasive stratification of atrial fibrosis. Furthermore, the model was not designed to replace invasive electroanatomical mapping but rather to provide a clinically meaningful, interpretable, and accessible approximation of atrial substrate characteristics. In this context, an R^2^ of 0.63 is acceptable for a first-step screening tool that could help guide further diagnostic or therapeutic decisions.

Previous studies have identified minimum LA volume as a more accurate predictor of atrial fibrosis than maximum volume [18,19]. However, concerns regarding its clinical utility have been raised due to limited reproducibility. Recent advances in imaging acquisition, particularly the emergence of 3D echocardiography, have helped overcome this limitation [20]. In our study, despite using two-dimensional techniques, the intra- and interobserver reproducibility of minimum volume measurements was acceptable; therefore, we find its use appropriate in clinical practice.

A decrease in passive LA emptying fraction has been proposed as an intermediate mechanism that contributes to elevated LA filling pressures and subsequent atrial remodelling. This functional impairment may either precede or coexist with structural changes and has been associated with AF recurrence, even after adjustment for LA late gadolinium enhancement on cardiac MRI [21]. According to a recent expert consensus on atrial cardiomyopathy, a total LA ejection fraction below 35% in sinus rhythm may be indicative of atrial systolic failure [3]. Our findings reinforce the clinical relevance of functional remodelling—particularly impaired passive emptying—as a meaningful surrogate marker of atrial fibrosis and atrial cardiomyopathy. In our cohort, approximately 50% of patients were in AF at the time of echocardiographic evaluation. Although subgroup analyses by rhythm status were conducted, the models exhibited poor predictive performance, and the analyses were not sufficiently powered to draw definitive conclusions. Nevertheless, these exploratory results suggest that rhythm at the time of echocardiography may influence model performance. Future studies with larger, rhythm-stratified cohorts should explore whether separate models for patients in sinus rhythm and AF can improve predictive accuracy.

Although our model exhibited an acceptable ability to predict MLAV, it did not effectively predict AF recurrence after PVI. Neither stratification by MLAV tertiles nor regression tree-defined groups showed significant associations with recurrence. This limitation may be explained by several factors: (1) heterogeneity in the small sample size across subgroups; (2) the potential influence of additional variables not accounted for, such as post-procedural diastolic changes or technical aspects related to the ablation procedure; and (3) the fact that a global voltage average may fail to capture localised areas of low voltage substrate that are mechanistically relevant for AF perpetuation. In the study by Ballesteros et al. [6], incorporating spatial distribution of voltage (Slope Voltage) significantly enhanced prediction of AF recurrence. Therefore, while MLAV remains a valuable surrogate of overall atrial fibrosis, our findings highlight its limited predictive value when used in isolation and underscore the need to complement it with more spatially sensitive markers.

Despite the lack of predictive value for AF recurrence, the ability to estimate MLAV non-invasively could still have relevant clinical implications. First, MLAV has been associated with the extent of atrial fibrosis, which is increasingly recognised as an independent risk factor for heart failure, stroke, and progression to persistent AF. Therefore, an approximate estimation of MLAV may contribute to early phenotyping of patients with AF, identifying those who could benefit from more intensive rhythm control strategies, closer monitoring, or additional imaging studies. Moreover, knowledge of the likely degree of atrial fibrosis before invasive procedures can inform procedural planning (e.g., the need for adjunctive substrate modification), guide patient counselling, and improve shared decision-making. Future studies combining MLAV estimation with other clinical and imaging biomarkers may yield more robust predictive tools with direct impact on patient outcomes.

### Limitations

This exploratory study supports the feasibility of developing a non-invasive model to estimate MLAV, providing a foundation for future research. However, the model is not yet suitable for clinical decision-making and should currently be considered a risk stratification tool. Several limitations must be acknowledged.

Methodological limitations include the single-centre design, moderate sample size, and absence of a formal a priori power calculation, all of which may limit generalisability. Internal validation using train–test split and cross-validation yielded encouraging results, but external validation is required. Advanced resampling techniques, such as bootstrapping, were not used and could improve robustness in future studies. CART was chosen for its interpretability and clinical applicability; while ensemble methods like Random Forest or XGBoost might achieve higher predictive performance, they were intentionally not applied to preserve model transparency and avoid overfitting. Future studies should explore and compare these approaches in larger, multicentre datasets.

Data and rhythm-related limitations are also relevant. Variables with >10% missingness were excluded, primarily due to physiological constraints rather than collection errors—for instance, parameters that could not be measured in patients in atrial fibrillation (e.g., pre-A LA volume) or when Doppler signals were suboptimal. No imputation was applied in this phase to preserve data integrity. Rhythm at the time of echocardiography may have influenced certain functional parameters; although subgroup analyses by rhythm status were performed, they showed inferior performance compared to the global model, likely due to reduced statistical power.

Clinical limitations include the model’s inability to predict AF recurrence after PVI. The inability to include contraction-dependent atrial metrics may have reduced the model’s comprehensiveness. Integration of additional predictors, such as circulating fibrosis biomarkers (e.g., Circulating Type I Collagen-Related Biomarkers), and exploration of rhythm-specific or ensemble-based models could improve predictive accuracy and clinical utility in future research.

## 5. Conclusions

In conclusion, we developed a novel ML-based model using a regression tree to predict MLAV, a surrogate marker of atrial fibrosis. The most powerful predictors identified were the minimum LA volume and passive atrial emptying fraction. Although further validation is required, our findings highlight the potential of clinical and echocardiography-based ML-models to enhance the non-invasive assessment of atrial fibrosis and, therefore, atrial cardiomyopathy.

## Figures and Tables

**Table 1:** Description and comparative analysis of clinical and electroanatomical variables.

Variables	Overall	“0.95” Group	“1.70” Group	“2.40” Group	*p*	Standardised Differences ^2^		
						0.95 vs. 1.70	0.95 vs. 2.40	1.7 vs. 2.40
*n*, %	113 (100.00)	72 (63.72)	19 (16.81)	22 (19.47)				
Mean voltage ± SD	1.41 ± 0.85	1.15 ± 0.71	1.63 ± 0.70	2.06 ± 1.01	<0.001 *	−0.68 (M)	−1.04 (L)	−0.49 (S)
Age, year ± SD	63.69 ± 10.19	65.63 ± 9.63	62.89 ± 9.60	58.05 ± 10.68	0.008 *	−0.19 (I)	0.08 (I)	0.27 (S)
Male sex, *n* (%)	82 (72.57)	54 (75.00)	13 (68.42)	15 (68.18)	0.744	0.06 (I)	0.07 (I)	0.01 (S)
Cardiovascular risk factors								
Tobacco								
Never smoker, *n* (%)	54 (47.79)	35 (48.61)	7 (36.84)	12 (54.55)	0.726	0.10 (S)	0.12 (S)	0.23 (S)
Previous smoker, *n* (%)	5 (4.42)	4 (5.56)	1 (5.26)	0 (0.00)				
Smoker, *n* (%)	54 (47.79)	33 (45.83)	11 (57.89)	10 (45.45)				
Hypertension, *n* (%)	63 (55.75)	43 (59.72)	10 (52.63)	10 (45.45)	0.477	0.06 (I)	0.12 (S)	0.07 (I)
Diabetes mellitus, *n* (%)	10 (8.84)	8 (11.11)	1 (5.26)	1 (4.55)	0.707	0.08 (I)	0.09 (I)	0.02 (I)
Dyslipidaemia, *n* (%)	51 (45.13)	37 (51.39)	5 (26.3)	9 (40.91)	0.134	0.2 (S)	0.09 (I)	0.15 (S)
Body mass index, kg/m^2^ ± SD	27.61 ± 3.97	27.93 ± 3.57	29.15 ± 4.23	25.24 ± 4.16	<0.003 *	0.06 (I)	−0.40 (S)	−0.41 (S)
Comorbidities								
Stroke, *n* (%)	9 (7.96)	8 (11.11)	1 (5.26)	0 (0.00)	0.205	0.08 (I)	0.17 (S)	0.17 (S)
Carotid artery disease, *n* (%)	4 (3.54)	4 (5.56)	0 (0.00)	0 (0.00)	0.454	0.11 (S)	0.12 (S)	-
COPD, *n* (%) ^1^	7 (6.19)	4 (5.56)	3 (15.79)	0 (0.00)	0.102	0.17 (S)	0.14 (S)	0.35 (M)
OSA, *n* (%) ^1^	12 (10.62)	9 (12.50)	2 (10.53)	1 (4.55)	0.613	0.03 (I)	0.11 (S)	0.12 (S)
CPAP use, *n* (%) ^1^	5 (4.42)	3 (4.17)	2 (10.53)	0 (0.00)	0.244	0.11 (S)	0.1 (S)	0.24 (S)
CKD, *n* (%) ^1^	9 (7.96)	8 (11.11)	0 (0.00)	1 (4.55)	0.370	0.16 (S)	0.09 (I)	0.15 (S)
Hypothyroidism, *n* (%)	15 (13.27)	10 (13.89)	3 (15.79)	2 (9.09)	0.850	0.02 (I)	0.06 (I)	0.1 (S)
Neoplasia, *n* (%)	16 (14.16)	10 (13.89)	3 (15.79)	3 (13.64)	-	0.02 (I)	<0.01 (I)	0.03 (I)
Previous cardiovascular history								
Heart failure, *n* (%)	16 (14.16)	13 (18.06)	2 (10.53)	1 (4.55)	0.283	0.08 (I)	0.16 (S)	0.12 (S)
Non-preserved LVEF, *n* (%) ^1^	12 (10.62)	9 (12.50)	2 (10.53)	1 (4.55)	0.613	0.03 (I)	0.11 (S)	0.12 (S)
Ischaemic heart disease, *n* (%)	10 (8.85)	9 (12.50)	1 (5.26)	0 (0.00)	0.214	0.01 (I)	0.18 (S)	0.17 (S)
Pacemaker/ICDs, *n* (%)	5 (4.42)	4 (5.56)	0 (0.00)	1 (4.55)	0.822	0.11 (S)	0.15 (S)	0.15 (S)
Medication								
Beta blockers, *n* (%)	61 (53.98)	41 (56.94)	9 (47.37)	11 (50.00)	0.694	0.08 (I)	0.06 (I)	0.03 (I)
Non-dihydropyridine antagonist, *n* (%)	3 (2.65)	3 (4.17)	0 (0.00)	0 (0.00)	-	0.10 (S)	0.10 (S)	-
Digoxin, *n* (%)	5 (4.42)	5 (6.94)	0 (0.00)	0 (0.00)	0.522	0.12 (S)	0.13 (S)	-
RAAS-related inhibitors, *n* (%) ^1^	54 (47.79)	37 (51.39)	6 (31.58)	7 (31.82)	0.237	0.11 (S)	0.16 (S)	0.04 (I)
Mineralocorticoid antagonist receptor, *n* (%)	7 (6.19)	7 (9.72)	0 (0.00)	0 (0.00)	0.119	0.15 (S)	0.16 (S)	-
Flecainide, *n* (%)	22 (19.47)	10 (13.89)	4 (21.05)	8 (36.36)	0.060	0.08 (I)	0.24 (S)	0.17 (S)
Propafenone, *n* (%)	2 (1.77)	2 (2.78)	0 (0.00)	0 (0.00)	-	0.08 (I)	0.08 (I)	-
Amiodarone, *n* (%)	13 (11.50)	10 (13.89)	2 (10.53)	1 (4.55)	0.577	0.04 (I)	0.12 (S)	0.12 (S)
AF characteristics								
Paroxysmal AF, *n* (%)	80 (70.80)	45 (62.50)	14 (73.68)	21 (95.42)	0.010 *	0.10 (S)	0.31 (M)	0.31 (M)
Typical atrial flutter, *n* (%)	29 (25.66)	17 (23.61)	8 (42.11)	4 (18.18)	0.188	0.17 (S)	0.06 (I)	0.26 (S)
EHRA ± SD ^1^	2 ± 0.37	1.9 ± 0.46	2 ± 0.00	2 ± 0.00	0.527	−0.30 (S)	−0.30 (S)	−0.01 (I)
CHA2DS2 VASc ± SD ^1^	1.82 ± 1.43	2.07 ± 1.54	1.68 ± 1.11	1.14 ± 1.08	0.024 *	0.29 (S)	0.70 (M)	0.49 (S)
Re-procedure, *n* (%)	21 (18.58)	14 (19.44)	3 (15.79)	4 (18.18)	0.934	0.04 (I)	0.01 (I)	0.03 (I)
AF diagnosis before PVI, months ± SD	40.99 ± 52.78	39.53 ± 54.63	60.74 ± 64.63	28.73 ± 25.87	0.351	−0.35 (S)	0.25 (S)	0.65 (M)
Follow-up after PVI, months ± SD	33.84 ± 16.40	35.01 ± 17.26	32.15 ± 13.38	31.45 ± 16.16	0.600	0.19 (I)	0.21 (S)	0.05 (I)
Recurrence, *n* (%)	52 (46.02)	37 (51.39)	10 (52.63)	5 (22.73)	0.041 *	<0.01 (I)	0.26 (S)	0.31 (M)

^1^ COPD, chronic obstructive pulmonary disease; OSA, obstructive sleep apnoea; CKD, chronic kidney disease; LVEF, left ventricular ejection fraction; RASS, renin angiotensin aldosterone system; PVI, pulmonary vein isolation; ICD, implantable cardioverter defibrillator; *n*, number of patients; *, statistically significant. ^2^ Effect sizes were calculated using Cohen’s *d* or Cramér’s *V*, as appropriate to the type of variable. For Cohen’s *d*: (I) negligible if <|0.20|; (S) small if 0.20–0.49; (M) moderate if 0.50–0.79; and (L) large if ≥0.80. For Cramér’s *V*: (I) negligible if <0.10; (S) small if 0.10–0.29; (M) moderate if 0.30–0.49; and (L) large if ≥0.50.

**Table 2:** Description and comparative analysis of the echocardiographic variables.

Variables	Overall	“0.95” Group	“1.70” Group	“2.40” Group	*p*	Standardised Differences ^2^		
						0.95 vs. 1.70	0.95 vs. 2.40	1.7 vs. 2.40
HR, bpm ± SD ^1^	67.47 ± 17.43	69.19 ± 18.98	62.63 ± 10.85	66.00 ± 16.37	0.431	0.42 (S)	0.18 (I)	−0.24 (S)
SBP, mm Hg ± SD ^1^	117.29 ± 16.72	116.64 ± 18.04	117.68 ± 16.1	119.14 ± 12.51	0.830	−0.06 (I)	−0.16 (I)	−0.10 (I)
DBP, mm Hg ± SD ^1^	75.35 ± 11.27	74.60 ± 10.93	76.16 ± 15.29	77.19 ± 8.00	0.660	−0.12 (I)	−0.27 (S)	−0.08 (I)
Body surface, m^2^ ± SD	1.93 ± 0.23	1.93 ± 0.21	1.99 ± 0.29	1.88 ± 0.21	0.297	−0.24 (S)	0.24 (S)	0.43 (S)
AF, *n* (%) ^1^	44 (38.94)	42 (58.33)	0.00 (0.00)	2 (9.09)	<0.001 *	0.41 (M)	0.42 (M)	0.21 (S)
LV dimensions and function parameters								
IVS, mm ± SD ^1^	1.05 ± 0.19	1.11 ± 0.19	0.98 ± 0.15	0.95 ± 0.17	<0.001 *	0.76 (M)	0.89 (L)	0.19 (I)
EDD, mm/m^2^ ± SD ^1^	2.52 ± 0.31	2.53 ± 0.31	2.46 ± 0.30	2.54 ± 0.34	0.662	0.23 (S)	−0.03 (I)	−0.25 (S)
LVPW, mm ± SD ^1^	1.12 ± 0.21	1.16 ± 0.22	1.03 ± 0.17	1.04 ± 0.17	0.013 *	0.66 (M)	0.61 (M)	−0.06 (I)
ESD, mm/m^2^ ± SD ^1^	1.71 ± 0.31	1.73 ± 0.31	1.70 ± 0.28	1.65 ± 0.36	0.554	0.10 (I)	0.24 (S)	0.16 (I)
EDLV volume, mL/m^2^ ± SD ^1^	45.31 ± 12.84	45.58 ± 13.58	41.07 ± 11.70	47.97 ± 10.90	0.233	0.36 (S)	−0.19 (I)	−0.61 (M)
ESLV volume, mL/m^2^ ± SD ^1^	18.60 ± 7.11	19.50 ± 7.62	15.42 ± 5.12	18.58 ± 6.41	0.090	0.63 (M)	0.13 (I)	−0.54 (M)
LVEF, % ± SD ^1^	58.38 ± 7.32	57.32 ± 7.60	60.37 ± 3.98	60.13 ± 8.24	0.022 *	−0.50 (M)	−0.35 (S)	0.04 (I)
Global longitudinal strain ± SD	18.72 ± 4.13	17.67 ± 4.23	19.82 ± 2.71	20.73 ± 3.85	0.007 *	−0.61 (M)	−0.76 (M)	−0.27 (S)
Mitral and tricuspid valve Doppler parameters								
E Vmax, cm/s ± SD	81.56 ± 21.19	86.72 ± 21.80	68.04 ± 15.31	76.38 ± 17.38	0.001 *	0.99 (L)	0.52 (M)	−0.51 (M)
A Vmax, cm/s ± SD	61.71 ± 18.50	57.72 ± 21.09	66.58 ± 18.95	63.05 ± 12.47	0.248	−0.44 (S)	−0.31(S)	0.22 (S)
E/A ratio ± SD	1.38 ± 0.62	1.69 ± 0.74	1.08 ± 0.35	1.20 ± 0.38	0.005 *	1.05 (L)	0.83 (L)	−0.33 (S)
e′, cm/s ± SD	10.48 ± 3.00	10.42 ± 2.61	9.83 ± 2.34	11.26 ± 4.39	0.506	0.24 (S)	−0.23 (S)	−041 (S)
E/e′ ratio ± SD	8.26 ± 3.34	8.81 ± 3.73	7.11 ± 1.83	7.43 ± 2.53	0.083	0.58 (M)	0.43 (S)	−0.14 (I)
a′ Vmax, cm/s ± SD	8.9 ± 2.98	7.63 ± 3.19	9.89 ± 2.91	9.84 ± 2.08	0.010 *	−0.74 (M)	−0.82 (L)	0.02 (I)
TR Vmax, m/s ± SD ^1^	2.31 ± 0.52	2.28 ± 0.52	1.98 ± 0.97	2.54 ± 0.33	0.225	0.39 (S)	−0.60 (M)	−0.77 (M)
LA diameters								
Anteroposterior, mm ± SD	42.32 ± 6.99	44.73 ± 6.44	39.58 ± 6.78	36.82 ± 4.64	<0.001 *	0.78 (M)	1.41 (L)	0.48 (S)
Maximum, mm ± SD	63.08 ± 8.54	65.93 ± 7.98	60.63 ± 8.73	55.95 ± 4.64	<0.001 *	0.63 (M)	1.53 (L)	0.67 (M)
Minimum, mm ± SD	44.70 ± 6.09	46.17 ± 6.05	40.26 ± 5.00	43.77 ± 5.12	<0.001 *	1.06 (L)	0.43 (S)	−0.69 (M)
LA volumes								
Maximum volume, mL/m^2^ ± SD	41.60 ± 11.97	47.28 ± 10.68	31.67 ± 8.36	32.87 ± 6.28	<0.001 *	1.63 (L)	1.64 (L)	−0.16 (I)
Minimum volume, mL/m^2^ ± SD	26.39 ± 12.48	33.53 ± 10.67	15.00 ± 3.06	14.83 ± 2.78	<0.001 *	2.36 (L)	2.40 (L)	0.06 (I)
preA volume, mL/m^2^ ± SD	27.09 ± 9.79	34.01 ± 10.76	22.74 ± 5.75	21.79 ± 4.51	<0.001 *	1.31 (L)	1.48 (L)	0.18 (I)
LA sphericity index (LASI)								
LASI ± SD	61.96 ± 18.40	63.22 ± 20.45	64.33 ± 15.15	56.21 ± 11.99	0.214	−0.06 (I)	0.42 (S)	0.59 (M)
LA function								
Total empty function, % ± SD	38.59 ± 16.55	29.72 ± 13.56	50.95 ± 12.37	54.5 ± 5.64	<0.001 *	−1.64 (L)	−2.39 (L)	−0.37 (S)
Passive empty function, % ± SD	57.70 ± 35.1	71.0 ± 36.38	24.21 ± 7.92	43.18 ± 14.17	<0.001 *	1.78 (L)	1.01 (L)	−1.65 (L)
Active empty function, % ± SD	30.13 ± 17.1	27.58 ± 23.88	32.63 ± 12.33	30.95 ± 9.30	0.040 *	−0.27 (S)	−0.19 (I)	0.15 (I)
LA strain								
Reserve, % ± SD	25.36 ±13.13	19.78 ±11.92	32.51 ± 8.68	36.30 ± 9.67	<0.001 *	−1.22 (L)	−1.52 (L)	−0.41 (S)
Conduit, % ± SD	17.25 ± 8.10	15.55 ± 7.72	17.51 ± 6.01	22.14 ± 8.96	<0.001 *	−0.28 (S)	−0.79 (M)	−0.61 (M)
Contraction, % ± SD	13.05 ± 5.64	10.34 ± 4.85	15.03 ± 6.21	14.88 ± 4.82	<0.001 *	−0.84 (L)	−0.94 (L)	0.03 (I)

^1^ HR, heart rate; SBP, systolic blood pressure; DBP, diastolic blood pressure; IVS, interventricular septum; EDD, end diastolic diameter; LVPW, left ventricular posterior wall; ESD, end systolic diameter; EDLV, end diastolic left ventricular; ESLV, end systolic left ventricular; *n*, number of patients; *, statistically significant. ^2^ Effect sizes were calculated using Cohen’s *d* or Cramér’s *V*, as appropriate to the type of variable. For Cohen’s *d*: (I) negligible if <|0.20|; (S) small if 0.20–0.49; (M) moderate if 0.50–0.79; and (L) large if ≥0.80. For Cramér’s *V*: (I) negligible if <0.10; (S) small if 0.10–0.29; (M) moderate if 0.30–0.49; and (L) large if ≥0.50.

## Data Availability

The data presented in this study are available on reasonable request from the corresponding author due to internal institutional policies that limit data dissemination for confidentiality and compliance reasons.

## Supplementary Materials

The following supporting information can be downloaded at: https://www.mdpi.com/article/10.3390/biomedicines13081917/s1, Table S1: Echocardiographic variables. Feasibility; Table S2: Echocardiographic variables. Intra- and interobserver reproducibility; Table S3: Echocardiographic and clinical analysed variables in the regression tree model; Table S4: Echocardiographic and clinical non-analysed variables in the regression tree model; Table S5: Post hoc between-group comparison; Table S6: Between-group comparison; Figure S1. Regression tree model for the prediction of mean left atrial voltage (MLAV) in patients in sinus rhythm; Figure S2. Regression tree model for the prediction of mean left atrial voltage (MLAV) in patients in atrial fibrillation.

## Abbreviations

The following abbreviations are used in this manuscript:

MLAV	Mean Left Atrial Voltage
ML	Machine Learning
AF	Atrial Fibrillation
PVI	Pulmonary Vein Isolation
uHDvM	Ultra-High-Density Voltage Mapping
CART	Classification and Regression Trees
AI	Artificial Intelligence
MRI	Magnetic Resonance
CUN	Clínica Universidad de Navarra
LV	Left Ventricle
LA	Left Atria
LAmax	LA Maximum Volume
LAmin	LA Minimum Volume
LApreA	LA preA Volume
LAEF	LA Total Emptying Fraction
LApEF	LA Passive Emptying Function
LAactEF	LA Active Emptying Function
LASres	LA Reservoir Strain
LAScd	LA Conduction Strain
LASct	LA Active Strain
