# Supplementary material for: Non-Invasive Prediction of Atrial Fibrosis Using a Regression Tree Model of Mean Left Atrial Voltage
Source: Biomedicines. 2025 Aug 6;13(8):1917. doi: 10.3390/biomedicines13081917 (PMC12383828; doi:10.3390/biomedicines13081917)
Supplement: Supplementary file 1 [file biomedicines-13-01917-s001.zip › biomedicines-3773544-supplementary.pdf]

SUPPLEMENTARY MATERIAL

TABLE S1. Echocardiographic variables. Feasibility.

Variables	Overall	Sinus rhythm	Atrial fibrillation	*P*-value	SD[1]
N (%)	113 (100)	69 (61)	44 (39)		
**LV dimensions and function parameters**					
IVS, n (%)	113 (100)	69 (100)	44 (100)		-
EDD, n (%)	113 (100)	69 (100)	44 (100)		-
LVPW, n (%)	113 (100)	69 (100)	44 (100)		-
ESD, n (%)	113 (100)	69 (100)	44 (100)		-
EDLV volume, n (%)	107 (95)	64 (93)	43 (98)	0.402	0.11 (S)
ESLV volume, n (%)	107 (95)	64 (93)	43 (98)	0.402	0.11 (S)
LVEF, n (%)	113 (100)	69 (100)	44 (100)		
Global longitudinal strain, n (%)	92 (81)	60 (87%)	32 (72%)	0.06	0.18 (S)
**Mitral and tricuspid valve Doppler parameters**					
E Vmax, n (%)	113 (100)	69 (100)	44 (100)		
A Vmax, n (%)	N.A.	69 (100)	N.A.		
e′, n (%)	111 (98)	67 (97)	44 (100)	0.26	0.11 (S)
E/e′, n (%)	108 (96)	65 (94)	43 (97)	0.65	0.08 (I)
A′ Vmax, n (%)	N.A.	62 (90)	N.A.		
TR Vmax, n (%)	55 (49)	27 (40)	28 (63)	**0.01***	0.24 (S)
**LA diameters**					
Anteroposterior, n (%)	113 (100)	69 (100)	44 (100)		
Maximum, n (%)	112 (99)	68 (99)	44 (100)	-	
Minimum, n (%)	112 (99)	68 (99)	44 (100)	-	
**LA volumes**					
Maximum volume, n (%)	110 (97)	67 (97)	43 (97)	-	
Minimum volume, n (%)	107 (95)	64 (93)	43 (97)	0.402	0.11 (S)
preA volume, n (%)	N.A.	67 (97)	N.A.		
**LA sphericity index**					
LA sphericity index, n (%)	110 (97)	67 (97)	43 (97)	-	
**LA function**					
Total empty fraction, n (%)	107 (95)	64 (93)	43 (97)	0.402	0.11 (S)
Passive empty fraction, n (%)	107 (95)	64 (93)	43 (97)	0.402	0.11 (S)
Active empty fraction, n (%)	N.A.	65 (57.5)	N.A.		

**LA strain**					
Reserve, n (%)	101 (90)	63 (91)	38 (86)	0.533	0.08 (I)
Conduit, n (%)	101 (90)	63 (91)	38 (86)	0.533	0.08 (I)
Contraction, n (%)	N.A.	64 (93)	N.A.		

Note: Differences in feasibility between patients in sinus rhythm and those in atrial fibrillation (AF) at the time echocardiography were determined. HR, heart rate; SBP, systolic blood pressure; DBP, diastolic blood pressure; IVS, interventricular septum; EDD, end-diastolic diameter; TSD, end-systolic diameter; LV, left ventricle; TR, tricuspid regurgitation; LA, left atrium; N.A., not appropriate; SD, standardized differences [1]Effect sizes were calculated using Cramér's V (I) negligible if < 0.10; (S) small if 0.10–0.29; (M) moderate if 0.30–0.49; and (L) large if ≥ 0.50**.**

TABLE S2. Echocardiographic variables. Intra- and inter-observer reproducibility.						
	Intra-observer			Inter-observer		
Variables	ICC	sup 95% CI	inf 95% CI	ICC	sup 95% CI	inf 95% CI
**LV dimensions and function parameters**						
LVEF	0.68	0.86	0.36	0.79	0.91	0.55
GLS	0.83	0.93	0.60	0.86	0.94	0.67
**Mitral and tricuspid valve Doppler parameters**						
E Vmax	0.95	0.88	0.98	0.91	0.78	0.96
A Vmax	0.98	0.94	0.99	0.96	0.85	0.99
e′	0.98	0.94	0.99	0.91	0.78	0.96
E/e′	0.98	0.96	0.99	0.97	0.92	0.97
A′ Vmax	0.99	0.98	0.99	0.96	0.86	0.99
TR Vmax	0.87	0.68	0.95	0.29	−0.29	0.72
**LA diameters**						
Anteroposterior	0.88	0.72	0.95	0.93	0.83	0.97
Maximum	0.92	0.80	0.97	0.91	0.78	0.96
Minimum	0.85	0.66	0.94	0.78	0.53	0.91
**LA volumes**						
Maximum volume	0.88	0.60	0.92	0.71	0.40	0.88
Minimum volume	0.89	0.75	0.96	0.81	0.58	0.92
preA volume	0.86	0.55	0.96	0.70	0.24	0.91
**LA function**						
Total empty fraction	0.71	0.41	0.87	0.67	0.33	0.86
Passive empty fraction	0.87	0.70	0.94	0.08	−0.37	0.50
Active empty fraction	0.71	0.18	0.93	0.25	−0.43	0.76
**LA sphericity index**						
LA sphericity index	0.88	0.60	0.92	0.71	0.40	0.88
**LA strain**						
Reservoir	0.94	0.85	0.98	0.91	0.80	0.97
Conduit	0.77	0.50	0.90	0.76	0.48	0.90
Contraction	0.84	0.44	0.97	0.98	0.93	0.99

Note: HR, heart rate; SBP, systolic blood pressure; DBP, diastolic blood pressure; IVS, interventricular septum; EDD, end-diastolic diameter; TSD, end-systolic diameter; LV, left ventricle; TR, tricuspid Regurgitation; LA, left atrium; N.A., not appropriate.

TABLE S3. Echocardiographic and clinical variables analysed.	
**Echocardiographic variable**	**Clinical variables**
LVEF	Age
E Vmax	Sex
e′	Tobacco
E/e′	Never smoker
Anteroposterior	Previous smoker
Maximum	Smoker
Minimum	Hypertension
Maximum volume	Diabetes mellitus
Minimum volume	Dyslipidaemia
Total empty fraction	Body mass index
Passive empty fraction	Stroke
LA sphericity index	Carotid artery disease
Reservoir Strain	COPD
Conduit Strain	OSA
	CPAP use
	CKD
	Hypothyroidism
	Neoplasia
	Heart failure
	Non-preserved LVEF
	LVEF
	Ischaemic heart disease
	Pacemaker/ICDs
	Beta blockers
	Non-dihydropyridine antagonist
	Digoxin
	RAAS-related inhibitors
	Mineralocorticoid antagonist receptor
	Flecainide
	Propafenone
	Amiodarone
	Paroxysmal AF
	Typical atrial flutter
	EHRA

	CHA2DS2 VASc
	Re-procedure
	Duration of AF

**TABLE S4. Echocardiographic and clinical variables no analysed.**	
**Echocardiographic variables**	
A Vmax	Contraction Strain
A′ Vmax	GLS
TR Vmax	
preA volume	
Active empty fraction	

**TABLE S5.** Regression tree subanalysis according to rhythm status			
	**Global**	**Atrial Fibrillation**	**Sinus Rhythm**
R^2	0.63	-1.10	-1.81
MSE	0.80	1.43	1.26
RMSE	0.75	0.98	0.89
MAE	0.90	1.20	1.12

Note: MSE, Mean Squared Error; RMSE, Root Mean Squared Error, MAE, Mean Absolute Error; R^2, Coefficient of Determination

Figure S1. Sinus Rhythm	Figure S2. Atrial Fibrillation
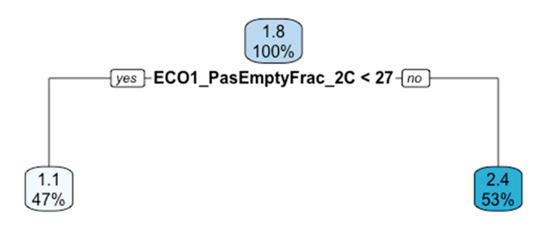	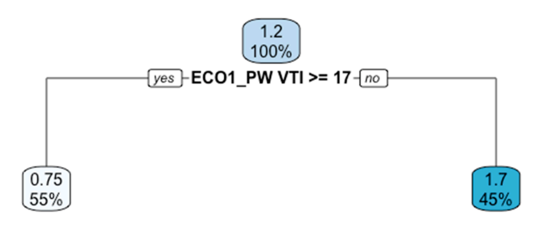

Figure S1. Regression tree model for the prediction of mean left atrial voltage (MLAV) in patients in sinus rhythm. The primary discriminative node was passive emptying fraction (PasEmptyFrac_2C), with a threshold value of 27%. Patients with a passive emptying fraction < 27% were assigned a lower estimated MLAV (1.1 mV), while those above this threshold had a higher predicted MLAV (2.4 mV). The model classified 47% and 53% of the population into the respective groups.

Figure S2. Regression tree model for the prediction of mean left atrial voltage (MLAV) in patients in atrial fibrillation. The primary discriminative node was left ventricular outflow tract (LVOT) diameter, with a threshold value of 17 mm. Patients with an LVOT diameter ≥ 17 mm were assigned a lower estimated MLAV (0.75 mV), while those with smaller diameters had a higher predicted MLAV (1.7 mV). The model classified 55% and 45% of the population into the respective groups.

TABLE S6. Between-group comparison.

Variable	P-value (Bonferroni)
**Clinical variables**	
Age, y	
‘0.95’ vs. ‘1.7’	0.853
‘0.95’ vs. ‘2.4’	0.006*
‘1.7’ vs. ‘2.4’	0.355

Body mass index, kg/m^2	
‘0.95’ vs. ‘1.7’	0.654
‘0.95’ vs. ‘2.4’	0.013*
‘1.7’ vs. ‘2.4’	0.004*
LVEF, %	
‘0.95’ vs. ‘1.7’	0.094
‘0.95’ vs. ‘2.4’	0.024*
‘1.7’ vs. ‘2.4’	-
Paroxysmal AF, n	
‘0.95’ vs. ‘1.7’	1.000
‘0.95’ vs. ‘2.4’	0.009
‘1.7’ vs. ‘2.4’	0.147
CHA2DS2 VASc	
‘0.95’ vs. ‘1.7’	0.861
‘0.95’ vs. ‘2.4’	0.022*
‘1.7’ vs. ‘2.4’	0.641
Recidive	
‘0.95’ vs. ‘1.7’	2.958
‘0.95’ vs. ‘2.4’	0.039*
‘1.7’ vs. ‘2.4’	0.141
AF at the time of echocardiography, n	
‘0.95’ vs. ‘1.7’	<0.001*
‘0.95’ vs. ‘2.4’	<0.001*
‘1.7’ vs. ‘2.4’	0.534
Interventricular septum, mm	
‘0.95’ vs. ‘1.7’	0.013*
‘0.95’ vs. ‘2.4’	0.001*
‘1.7’ vs. ‘2.4’	0.950
Left ventricular posterior wall, mm	
‘0.95’ vs. ‘1.7’	0.061
‘0.95’ vs. ‘2.4’	0.067
‘1.7’ vs. ‘2.4’	-
LVEF, %	
‘0.95’ vs. ‘1.7’	0.094
‘0.95’ vs. ‘2.4’	0.024*
‘1.7’ vs. ‘2.4’	-

Global longitudinal strain	
‘0.95’ vs. ‘1.7’	0.191
‘0.95’ vs. ‘2.4’	0.009*
‘1.7’ vs. ‘2.4’	-
E Vmax, cm/s	
‘0.95’ vs. ‘1.7’	0.001*
‘0.95’ vs. ‘2.4’	0.100
‘1.7’ vs. ‘2.4’	0.563
E/A ratio	
‘0.95’ vs. ‘1.7’	0.003*
‘0.95’ vs. ‘2.4’	0.045*
‘1.7’ vs. ‘2.4’	0.5620*
a′ Vmax, cm/s	
‘0.95’ vs. ‘1.7’	0.034*
‘0.95’ vs. ‘2.4’	0.035*
‘1.7’ vs. ‘2.4’	-
Anteroposterior diameter, mm	
‘0.95’ vs. ‘1.7’	0.005*
‘0.95’ vs. ‘2.4’	0.000*
‘1.7’ vs. ‘2.4’	0.477
Maximum diameter, mm	
‘0.95’ vs. ‘1.7’	0.039*
‘0.95’ vs. ‘2.4’	<0.001*
‘1.7’ vs. ‘2.4’	0.082
Minimum diameter, mm	
‘0.95’ vs. ‘1.7’	<0.001*
‘0.95’ vs. ‘2.4’	0.265
‘1.7’ vs. ‘2.4’	0.158
Maximum volume, mL/m^2	
‘0.95’ vs. ‘1.7’	<0.001*
‘0.95’ vs. ‘2.4’	<0.001*
‘1.7’ vs. ‘2.4’	-
Minimum volume, mL/m^2	
‘0.95’ vs. ‘1.7’	<0.001*
‘0.95’ vs. ‘2.4’	<0.001*
‘1.7’ vs. ‘2.4’	-

preA volume, mL/m^2	
‘0.95’ vs. ‘1.7’	<0.001*
‘0.95’ vs. ‘2.4’	<0.001*
‘1.7’ vs. ‘2.4’	-
Total empty function, %	
‘0.95’ vs. ‘1.7’	<0.001*
‘0.95’ vs. ‘2.4’	<0.001*
‘1.7’ vs. ‘2.4’	0.794
Passive empty function, %	
‘0.95’ vs. ‘1.7’	<0.001*
‘0.95’ vs. ‘2.4’	0.176
‘1.7’ vs. ‘2.4’	<0.001*
Active empty function, %	
‘0.95’ vs. ‘1.7’	0.030*
‘0.95’ vs. ‘2.4’	0.081*
‘1.7’ vs. ‘2.4’	-
Reserve, %	
‘0.95’ vs. ‘1.7’	0.000*
‘0.95’ vs. ‘2.4’	0.000*
‘1.7’ vs. ‘2.4’	0.881
Conduit, %	
‘0.95’ vs. ‘1.7’	1.00
‘0.95’ vs. ‘2.4’	0.003*
‘1.7’ vs. ‘2.4’	0.209
Contraction, %	
‘0.95’ vs. ‘1.7’	<0.001*
‘0.95’ vs. ‘2.4’	0.265
‘1.7’ vs. ‘2.4’	0.158
